# Expanding the Knowledge of KIF1A-Dependent Disorders to a Group of Polish Patients

**DOI:** 10.3390/genes14050972

**Published:** 2023-04-25

**Authors:** Justyna Paprocka, Aleksandra Jezela-Stanek, Robert Śmigiel, Anna Walczak, Hanna Mierzewska, Anna Kutkowska-Kaźmierczak, Rafał Płoski, Ewa Emich-Widera, Barbara Steinborn

**Affiliations:** 1 Department of Pediatric Neurology, Faculty of Medical Sciences, Medical University of Silesia, 40-752 Katowice, Poland; 2 Department of Genetics and Clinical Immunology, National Institute of Tuberculosis and Lung Diseases, 01-138 Warsaw, Poland; 3 Department of Family and Pediatric Nursing, Wroclaw Medical University, 50-368 Wroclaw, Poland; 4 Department of Medical Genetics, Warsaw Medical University, 02-091 Warsaw, Poland; 5 Department of Child and Adolescent Neurology, Institute of Mother and Child, 01- 211 Warsaw, Poland; 6 Department of Medical Genetics, Institute of Mother and Child, 01-211 Warsaw, Poland; 7 Department of Developmental Neurology, Poznan University of Medical Sciences, 61-701 Poznan, Poland

**Keywords:** *KIF1A*, neurodegeneration, SPG30, HSN2C, NESCAV, children

## Abstract

Background: *KIF1A* (kinesin family member 1A)-related disorders encompass a variety of diseases. *KIF1A* variants are responsible for autosomal recessive and dominant spastic paraplegia 30 (SPG, OMIM610357), autosomal recessive hereditary sensory and autonomic neuropathy type 2 (HSN2C, OMIM614213), and autosomal dominant neurodegeneration and spasticity with or without cerebellar atrophy or cortical visual impairment (NESCAV syndrome), formerly named mental retardation type 9 (MRD9) (OMIM614255). *KIF1A* variants have also been occasionally linked with progressive encephalopathy with brain atrophy, progressive neurodegeneration, PEHO-like syndrome (progressive encephalopathy with edema, hypsarrhythmia, optic atrophy), and Rett-like syndrome. Materials and Methods: The first Polish patients with confirmed heterozygous pathogenic and potentially pathogenic *KIF1A* variants were analyzed. All the patients were of Caucasian origin. Five patients were females, and four were males (female-to-male ratio = 1.25). The age of onset of the disease ranged from 6 weeks to 2 years. Results: Exome sequencing identified three novel variants. Variant c.442G>A was described in the ClinVar database as likely pathogenic. The other two novel variants, c.609G>C; p.(Arg203Ser) and c.218T>G, p.(Val73Gly), were not recorded in ClinVar. Conclusions: The authors underlined the difficulties in classifying particular syndromes due to non-specific and overlapping signs and symptoms, sometimes observed only temporarily.

## 1. Introduction

The *KIF1A* (kinesin family member 1A) gene is located on chromosome 2q37.3 and is expressed mainly in the brain and spinal cord. The gene codes the KIF1A protein—one of the kinesin superfamilies of microtubular-dependent molecular motors involved in retrograde axonal transport of dense-core vesicles [1,2]. One of the neuropeptides transported in those vesicles is the brain-derived neurotrophic factor (BDNF) [2]. Because of that, KIF1A may play a crucial role in neuronal development, synaptic maturation, and function [3]. Its mutations have been associated with three different disorders in OMIM (https://www.omim.org 25 March 2023), all of which include severe neurological symptoms.

Pathogenic variants in the *KIF1A* gene are responsible mainly for three phenotypes—autosomal recessive and dominant spastic paraplegia 30 (SPG30, OMIM 610357), autosomal recessive hereditary sensory and autonomic neuropathy type 2 (HSN2C, OMIM 614213), and autosomal dominant neurodegeneration and spasticity with or without cerebellar atrophy or cortical visual impairment syndrome NESCAVS (OMIM 614255). Occasionally, *KIF1A* variants have also been linked with progressive encephalopathy with brain atrophy, progressive neurodegeneration, PEHO-like syndrome (progressive encephalopathy with edema, hypsarrhythmia, optic atrophy), and Rett-like syndrome. 

Both autosomal dominant or recessive forms of SPG30 and autosomal recessive HSN2C are relatively milder forms with onset in the first decade of life. NESCAV syndrome (OMIM 614255), formerly known as autosomal dominant intellectual disability 9 (MRD9), is a severe neurodegenerative disorder. Its clinical presentation may vary, but it is characterized by cognitive impairment and progressive spasticity, predominantly in the lower limbs. Global development is usually profoundly delayed, with intellectual disability, speech delay or absence, and behavioral problems. Cerebellar atrophy is frequently present in NESCAV patients, usually manifesting with ataxia. Ophthalmologic assessment may present optic nerve atrophy, cortical visual impairment, and nystagmus. Patients may also be diagnosed with peripheral sensorimotor axonal neuropathy [4]. Most identified *KIF1A* variants associated with NESCAV syndrome were heterozygous and occurred de novo [5,6,7].

The authors present the first group of Polish patients diagnosed with known and *novel KIF1A* heterozygous mutations and analyze their genotypes and clinical phenotypes in comparison with previous reports. 

## 2. Materials and Methods

### Patients

The authors present the first nine Polish patients with confirmed heterozygous pathogenic and potentially pathogenic *KIF1A* variants. All the patients were of Caucasian origin. A consanguineous background was absent. Five patients were females, and four were males (female-to-male ratio = 1.25). Age of onset of the disease was set at the appearance of the first symptoms and ranged from 6 weeks to 2 years. A spectrum of clinical features presented by the patients is summarized in Table 1 and Table 2. Signed written informed consent for genetic analysis was obtained from all the parents of individuals enrolled in the study.

## 3. Results

### Molecular Study

Whole-exome sequencing (WES) was performed in all nine Polish patients to search for the cause of complex neurodevelopmental symptoms. SureSelectXT Human All Exon kit (Agilent, Agilent Technologies, Santa Clara, CA, USA) was used according to the manufacturer’s instructions. The enriched library was paired-end sequenced (2 × 100 bp) on a HiSeq 1500 (Illumina, San Diego, CA, USA) to the mean depth of 85×. Raw data analysis and variant prioritization were performed as previously described [8,9]. Variants considered as causative were validated using DNA samples from the proband and proband’s parents by amplicon deep sequencing (ADS) performed with a Nextera XT Kit (Illumina) and paired-end sequenced (2 × 100 bp) on a HiSeq 1500 (Illumina, San Diego, CA, USA). Table 2 shows the variants of the *KIF1A* gene (LRG_367; NM_001244008.1) detected in the WES tests.

Exome sequencing identified seven different de novo variants and one variant which appeared to be inherited from the mosaic parent (paternal origin) from nine independent patients. All the identified variants were absent in the gnomAD database. The mosaic father of patient 5 was unaffected. In addition, we tested semen collected from the father. Variant c.946C>T was detected, using the ADS method, in patient 5’s father’s blood at VAF (variant allele frequency) 21% (coverage: 8788x) and in semen at 22% (coverage: 2966x). Every variant found in our patients causes a change in one amino acid (missense type). Among the nine patients, one variant, previously described, was detected in two patients: c.815A>G, p.(Asn272Ser). The remaining seven variants include four already reported: c.38G>A, p.(Arg13His); c.646C>T, p.(Arg216Cys); c.946C>T, (p.Arg316Trp); and c.296C>T; p.(Thr99Met). Three novel variants were identified. Variant c.442G>A is described in the ClinVar database as likely pathogenic. The other two novel variants, c.609G>C; p.(Arg203Ser) and c.218T>G, p.(Val73Gly), do not have any record in ClinVar. In silco prediction by SIFT revealed these three variants as pathogenic (data obtained from the varsome website: https://varsome.com/, accessed on 1 March 2022) [10,11,12,13]. All three novel variants are located within highly conserved regions of the kinesin motor domain.

## 4. Discussion

### 4.1. KIF1A-Related Phenotypes

The KIF1A-related recessive disease variants of the motor domain 6.7 may have different consequences on the protein’s function than the dominant variants, which could explain why they do not cause disease in heterozygous carriers. Structural modeling suggests that the dominant disease variants affect ATP binding, γ-phosphate release, or microtubule binding. Based on structural analyses, the recessive variants were predicted to disrupt the back door structure or the neck linker between the motor domain and cargo-binding regions, respectively [1,2,14,15]. Functional experimental studies suggest that recessive variants impair motor function to a lesser extent than dominant ones [1,2,14,15]. Thus, it is usually challenging to predict the disease progression, even in the same families, and KIF1A-related disorders can best be thought of as a spectrum of diseases, ranging from mild symptoms to severe, life-threatening complications, including the main clinical spectra such as neurodegeneration and spasticity with or without cerebellar atrophy or cortical visual impairment (NESCAV syndrome; OMIM 614255), formerly mental retardation, autosomal dominant 9 (MRD 9) and hereditary sensory neuropathy type IIC (HSN2C; MIM 614213), as well as autosomal recessive and dominant spastic paraplegia 30 (SPG30; OMIM 610357).

The first reported phenotypes of *KIF1A* mutation included pure hereditary spastic paraplegia (HSP). Between the clinical spectra, HSP is among the most common KIF1A-related pathology. HSP includes over 80 genetic types that are designated SPG (spastic paraplegia), numbered in the order of their discovery. Spastic paraplegias may also be caused by genes not typically connected with SP [16,17,18,19]. In general, HSP prevalence is estimated as 3–10/100,000. Elsayed et al. proposed interesting hints for HSP diagnosis [20]. According to Elsayed et al., HSP could be divided into two main groups: complex HSP (AD inheritance: SPG4, SPG6, SPG8, SPG10, SPG12, SPG13, SPG19, SPG31, SPG33, SPG41, SPG42, SPG73, SPG80; AR inheritance: SPG5A, SPG11, SPG15, SPG24, SPG27, SPG28, SPG45/65 (NT5C2), SPG56, SPG57, SPG58, SPG62, SPG76, SPG77, SPG80; AR/AD: SPG3A, SPG7, SPG9, SPG18, SPG30, SPG72; X-linked recessive inheritance: SPG16, SPG34) and pure HSP (AD inheritance: SPG12, SPG13, SPG19, SPG41, SPG42; AR inheritance: SPG24, SPG62, SPG83; X-linked recessive inheritance: SPG34). HSP can include cognitive impairment/intellectual disability, peripheral neuropathy (with/without amyotrophy), cerebellar signs (with/without evidence of cerebellar atrophy on brain MRI), extrapyramidal signs, optic atrophy, cataract, strabismus, retinal/macular degeneration, anarthria, seizures/epilepsy, stereotypic laughter, microcephaly complicated with short stature, developmental delay, skeletal deformities, hypogonadism, and infertility [20].

The last of the KIF1A-related syndromes is spastic paraplegia 30 (SPG30) (OMIM610357). It has been associated with both autosomal dominant and autosomal recessive transmission patterns. Some patients have also been identified as having de novo heterozygous mutations [10,11,12,13,14,16,17,18,19]. SPG30 is characterized by slowly progressive spastic paraplegia with onset in adolescence or adulthood. Although in some cases SPG30 affects only the locomotor system with lower limb spasticity and pyramidal signs, other neurological problems may occur [21]. The main, practically omnipresent additional sign in complicated SPG30 is intellectual disability (ID) ranging from severe (more often) to mild. The reported clinical spectrum covers microcephaly, epilepsy, optic atrophy, ataxia, axonal neuropathy, dystonia, brain MRI abnormalities (cerebral and/or cerebellar atrophy, hypogenesis/thinning of corpus callosum, white matter lesion), epilepsy, optic atrophy, blindness of central origin, axonal neuropathy, axial hypotony, athetosis, dystonia HSP which may be complicated by cerebellar atrophy, intellectual disability and/or axonal neuropathy, and severe neonatal presentation with progressive encephalopathy with brain atrophy [6,9,21,22] Recently, Montenegro-Garreaud reported evidence supporting the association of hip subluxation, dystonia, and gelastic epilepsy with KIF1A dysfunction [23].

Klebe et al. reported frequent sphincter disturbances, mild ataxia, and sensory deficit in SPG30 patients [3], whereas other studies indicate that cognition is usually normal; however, some studies show the presence of mild intellectual disability and learning difficulties [17,18]. 

Pure SPG30 resembles SPG3 or SPG4, with a slow course and an age of onset between one year and seventy years. Most of the described patients seem to be diagnosed with cHSCP. With the clinical data of the Polish cohort presented here, we can support the importance of *KIF1A* variants in the development of spastic paraplegia. 

*KIF1A* mutations may also result in hereditary sensory neuropathy type IIC (HSN2C) (MIM #614213) [4,14,15]. Given the mentioned KIF1A function, it may play a critical role in the development of axonal neuropathies resulting from impaired axonal transport. It was described in 2011 by Rivière et al. as a progressive distal sensory loss leading to ulceration and amputation of fingers and toes [4]. Position and vibration senses were impaired the most with accompanying distal motor deterioration. HSN2C is inherited with an autosomal recessive pattern, and its first symptoms are present in the first decade [14,21,22].

### 4.2. Molecular Characteristics and Clinical Correlation of Polish Patients

Analyzing the clinical symptoms of our patients, we noticed that the phenotypic variation in KIF1A mutations is much broader than previously described. We had difficulty classifying them among individual phenotypes due to overlapped clinical pictures and dynamically changing phenotypes. Our patients presented mainly a severe phenotype. The onset of the symptoms was observed in early infancy. Six of them were classified as NESCAVS. Older patients had an abnormal MRI with brain atrophy. The overlapped clinical picture between KIF1-related disorders and Rett-like syndrome (psychomotor retardation/arrest, abnormal breathing pattern, stereotyped hand movements) may be due to the common target gene, neurotrophin-brain-derived neurotrophic factor (BDNF) [9]. 

Three of our patients presented with novel *KIF1A* variants: c.442G>A/p.(Glu148Lys); c.609G>C/p.(Arg203Ser); and c.218T>G/p.(Val73Gly). Based on the literature data, among the patients with the same *KIF1A* mutation variant, there is huge variability in disease progression and severity of symptoms. The KIF1A gene is in the cytogenetic 2q37.3 band. According to the Human Mutation Database, 103 variants have been identified in the *KIF1A* gene (accessed 25 March 2022) [10,11,12,13]. Comparisons of our patients with the available literature data are shown in Table 3, Table 4 and Table 5. KIF1A-related disorders probably remain underdiagnosed because of the dominant relation with HSP and less known coincidence with a multisystem and progressive course with upper motor neuron dysfunction and extrapyramidal signs with neuropathy [24,25,26,27,28,29,30,31]. 

Nicita et al. performed genotype–phenotype correlations in 19 patients aged 3–65 years, including 14 children [14]. The patients were divided into 2 groups: group 1 with a complex phenotype: dominant pyramidal signs and additional features: epilepsy, ataxia, peripheral neuropathy, and optic nerve atrophy, and group 2 with an early-onset or congenital ataxic phenotype. In our group, all patients presented at the beginning with psychomotor retardation [14]. Conversely to the group described by Nicita et al., most of our patients had normal MRI findings (7/9). Considering the age of the children, we can speculate that the progression of cerebellar and cerebral atrophy may appear with time. According to the literature data, a pure cerebellar ataxia phenotype has been reported very rarely. Epilepsy was diagnosed in four patients. The semiology of seizures varied, resulting in differences in the AEDs used. According to the observation of our patients with *KIF1A* mutations, epilepsy may present in age-dependent stages. In the onset phase, epilepsy manifests with a very high activity, sometimes before the development of other recognizable clinical features. With age, like in many genetic syndromes, there is a tendency for seizures to decrease up to cessation. 

The detected variants in the *KIF1A* gene in our group of patients are consistent with the autosomal dominant mode of inheritance. The correlation of genotype–phenotype is hard in this case, as was previously reported, and because of that, dominant forms of *KIF1A*-related disorders are characterized by a more complex phenotype, wider spectrum of symptoms, and higher severity and age of onset [14]. Table 3, Table 4, Table 5 and Table 6 compare the phenotypes of our patients with those previously reported. It is worth highlighting how variable the symptoms are. 

Interestingly, contrary to the literature, ataxia was not observed at the last assessment of our patients [24,25]. In one individual (patient 1), cerebellar atrophy was noted in neuroimaging, and he presented with dystonia. It is very important to note that the diagnosis in our patients may change with age as symptoms of HSP increase, and the clinical picture may correspond to HSP with neuropathy more than NESCAV. Therefore, it is important to differentiate between clinical forms of KIF1A-related disorders. Moreover, further detailed clinical and genetic characterization of patients should be performed with broad international cooperation.

## Figures and Tables

**Table 1:** Clinical features of patients included in the study.

Number of Patient, Age, and Sex	Patient 1 11 yrs, Male	Patient 2 10 yrs, Female	Patient 3 9 yrs, Male	Patient 4 6,5 yrs, Male	Patient 5 6 yrs, Female	Patient 6 5 yrs, Female	Patient 7 5 yrs, Female	Patient 8 3 yrs, Male	Patient 9 2 yrs 7 mo, Male
Family history	Non-remarkable	Non-remarkable	Hashimoto disease—mother	Celiac disease	Down syndrome in aunt’s daughter	Diabetes mellitus, asthma	Fetal hypotrophy, two-vessel umbilical cord	Non-remarkable	Non-remarkable
Gestation, delivery period	GII, DII, vaginal delivery, birth weight: 3830 g, birth HC: 34 cm, Apgar: 10 points	GII, DII, 41 weeks, vaginal delivery, birth weight: 3430 g, Apgar: 10 points	GII, DII, vaginal delivery, 40 weeks, poor fetal movements. birth weight: 3870 g, birth HC: 36 cm, Apgar: 10 points	GI, DI, 40 weeks, caesarian section, birth weight: 3440 g, birth HC: 34 cm, Apgar: 10 points	GI (diabetes mellitus—insulin therapy, risk of preterm delivery), DI, 40 weeks, vaginal delivery, birth weight: 2550 g, birth HC: 31 cm, Apgar: 10 points, short umbilical cord	GI (urinary tract infections, hypothyroidismEuthyrox), DI, 39 weeks, birth weight: 3550 g, birth HC: 35 cm, Apgar: 10 points	GI, DI, 38 weeks, vaginal delivery, birth weight: 2200 g, birth HC: 29 cm, Apgar: 10 points	GI, DI, 38 weeks, vaginal delivery, birth weight: 3400 g, birth HC: 32.5 cm, Apgar: 10 points	GI, DI, vaginal delivery, 39 weeks, birth weight: 3500 g, birth HC: 34 cm, Apgar 10 points
First abnormalities in child’s development	1.5 mo—urosepsis 6 mo—developmental delay	2 yrs—gait abnormalities, delayed speech development	6 weeks—hypotonia, asymmetry, hand tremor	4–6 mo	8 mo—West syndrome	6 mo	Postnatal hypotrophy, microcephaly	6 mo	6–7 mo—developmental arrest and regression, vision problems
Psychomotor development	Standing at 19 mo, walking with support, simple phrases	24 mo—walking disorders	Sitting at 14 mo, walking at 3 yrs, simple words	Rolling over—5/6 mo, sitting—12 mo, crawling—2.5 yrs, standing—18 mo without walking, first words—1.5 yrs, simple phrases	Sitting—24 mo, walking with support—40 mo	Not able to roll over, simple syllables	Sitting—10 mo	Without independent sitting	Rolling over—7/8 mo, without independent sitting
Epilepsy	Left-sided seizures at the age of 3.5 yrs	Not applicable	6 yrs—epilepsy with continuous discharges in sleep	Not applicable	Epileptic spasms (8 mo–16 mo), upward eye rotation—15 mo	Polymorphic seizures	Not applicable	Not applicable	Not applicable
Antiepileptic treatment	Valproic acid, at present without treatment	Not applicable	Valproic acid, clobazam, CBD oil	Not applicable	Vigabatrin, valproic acid, levetiracetam, topiramate, clobazam, ethosuximide, ketogenic diet, CBD oil since 01/2018 without drugs apart from ketogenic diet with complete seizure control	Valproic acid, vigabatrin, levetiracetam, lacosamide (antiepileptic treatment was stopped at the age of 2.5 yrs)	Not applicable	Not applicable	Not applicable
Spastic paraplegia	+	+	-	+	+	+	+	+	+
Neuropathy	-	-	-	+	-	+	-	+	+
Physical examination	Higher risk of hip subluxation, open-mouth appearance, wide space between first teeth, narrow upper lip, mildly pectus carinatum	-	Single cafe-au-lait spot	Hip subluxation	-	-	-	Hip subluxation	Hip subluxation
Dystonia/Dyskinesia	+	-	+	-	+	-	-	+	+
Psychological assessment	Moderate intellectual disability	2.5 yrs—speech development slightly delayed	IR = 50, single words	No speech	Single words	No speech	2.5 yrs—speech development slightly delayed	No speech	No speech
MRI	Atrophy of cerebellar hemispheres, hypoplasia of lower part of cerebellar vermis, mild corpus callosum hypoplasia	Normal	Normal at 15 mo, mild brain atrophy at 7 yrs	8 mo, 18 mo—normal	Mild cerebral atrophy	Normal	Normal	Normal	Normal
EEG	Normal	Normal	Normal background activity, continuous paroxysmal discharges in sleep	Normal background activity, single and series of generalized or localized in left hemispheres, delta and sharp waves	Normal	Normal	Normal	Abnormal, without epileptic activity	Abnormal, without epileptic activity
Ophthalmologic examination	Strabismus, astigmatism, hypermetropia	Normal	Normal	Normal	1.5 yrs: +7.5D bilaterally, 2 yrs: +4.0D bilaterally	Normal	Normal	Cortical visual disturbances	Optic nerve atrophy
Hearing	Normal	Normal	Normal	Normal	Normal	Normal	Normal	Normal	Normal
Age of genetic study	9 yrs	7 yrs	4 yrs	3 yrs	3.5 yrs	2.5 yrs	14 mo	16 mo	2 yrs
Sleep problems	No	No	No	No	Yes	No	No	No	No
Dominant clinical picture	cHSCP	NESCAV	NESCAV	cHSCP	cHSCP	NESCAV	NESCAV	NESCAV	cHSCP

**Table 2:** Variants of *KIF1A* (LRG_367; NM_001244008.1) gene detected in WES tests of patients included in the study.

	Patient 1 * 11 yrs	Patient 2 10 yrs	Patient 3 9 yrs	Patient 4 6,5 yrs	Patient 5 6 yrs	Patient 6 5 yrs	Patient 7 7 yrs	Patient 8 * 3 yrs	Patient 9 * 2 yrs 7 mo
Confirmation and family testing method	ADS (amplicon deep sequencing)	Sanger sequencing	ADS	Sanger sequencing	ADS	ADS	Sanger sequencing	ADS	ADS
Identified variant (hg38)	2:240786501-C>T; c.442G>A; p.(Glu148Lys) de novo	2:240797715-C>T; c.38G>A; p.(Arg13His) de novo	2:240785063-G>A; c.646C>T, p.(Arg216Cys) de novo	chr2:240783093-T>C; c.815A>G, p.(Asn272Ser)	2:240775863-G>A, c.946C>T, p.(Arg316Trp), paternal mosaicism	2:240788118-G>A; c.296C>T; p.(Thr99Met) de novo	chr2:240783093-T>C; c.815A>G, p.(Asn272Ser)	2:240785100-C>G; c.609G>C; p.(Arg203Ser) de novo	2:240788196-A>C; c.218T>G, p.(Val73Gly) de novo
ClinVar	Likely pathogenic	Pathogenic	Pathogenic	Pathogenic	Pathogenic	Pathogenic	Pathogenic	No data	No data
SIFT	Pathogenic	Pathogenic	Pathogenic	Uncertain	Pathogenic	Pathogenic	Uncertain	Pathogenic	Pathogenic

* Novel variants.

**Table 3:** Patient 5 with c.946C>T mutation and available literature data [8,14].

		Our Patient	Lee at al., 2015 [8]	Nicita et al., 2020 [16]	
Patient		Patient 5	10	10	11
Phenotype		cHSCP	?	cHSCP	cHSCP
Age		6 y	10 y	5y	10 y
Gender		F	F	M	M
Mutation, inheritance		c.946C>T (parental, mosaicism)	c.946C>T p.R316W, de novo	c.946C>T	c.946C>T. de novo
Age of onset		8 mo	N/A	6–8 mo	<1 mo
Global development		Moderate	Mild	Severe	Severe
Scoliosis		-	N/A	+	-
Microcephaly		-	-	-	+
Epilepsy, seizures		Epileptic spasms (8 mo–16 mo), upward eye rotation (15 mo)	-	Focal seizures	-
EEG		Normal background activity (10/17), single paroxysmal and series delta and sharp waves in left hemisphere	N/A	Multifocal abnormalities	Multifocal abnormalities
Antiepileptic treatment		Vigabatrin, valproic acid, levetiracetam, topiramate, clobazam, ethosuximide, ketogenic diet, CBD oil since 2.5 years, only ketogenic diet, and complete seizure control	N/A	N/A	N/A
Spastic paraparesis		+	+	+	Spastic tetraparesis
Axial hypotonia		+	N/A	_	+
Cerebellar signs		-	Ataxia	+	+
Neuropathy		-	-	Intermediate sensory–motor neuropathy	Sensory neuropathy
Optic nerve atrophy		-	+	+	+
Other neurological dysfunction		-	-	Extrapyramidal signs	-
MRI	Cerebellar atrophy	-	+	Mild	+
	Cerebral atrophy	-	-	+	-
	Dilatation of lateral ventricles	+	-	-	-
Course		Non-progressive	N/A	Non-progressive	Regressive

**Table 4:** Patients 4 and 7 with c.815A>G mutation and available literature data [14].

		**Our Patients**		**Nicita et al., 2020** [16]
Patient		Patient 4	Patient 7	Patient 7
Phenotype		cHSCP	NESCAV	Congenital-onset (cHSCP—hereditary spastic paraparesis)
Age		6.5 yrs	5 yrs	13 yrs
Gender		M	F	M
Mutation, inheritance		c.815A>G, de novo	c.815A>G, de novo	c.815A>G, de novo
Age of onset		4–6 mo	12 mo	5 mo
Global development delay		Moderate	Moderate	Severe
Epilepsy, seizures		-	-	Febrile seizures
EEG		Generalized abnormalities (spike–slow waves, sharp and slow waves)	Normal	Bilateral anomalies
Antiepileptic treatment		N/A	N/A	N/A
Spastic paraplegia		Spastic paraparesis	Spastic paraparesis	Spastic tetraparesis
Axial hypotonia		+	+	-
Cerebellar signs		Ataxia	-	-
Neuropathy		-	-	-
Optic neuropathy		+	-	+
Other neurological dysfunction		-	-	Stereotyped movements
MRI			Normal	
	Cerebellar atrophy	+	-	+
	Cerebral atrophy	-	-	-
	Dilatation of lateral ventricles	-	-	-
	Thin corpus callosum	-	-	+

**Table 5:** Patient 6 with mutation c.296C>T and available literature data [5,6,8,21].

		Our Patient	Hamdan et al., 2011 [29]	Okamoto et al., 2014 [6]	Lee et al., 2015 [8]		Langlois et al., 2016 [5]	Nicita et al., 2020 [16]		Nieh et al., 2015 [9]	
		Patient 6	Patient 7		Patient 1	Patient 2	Patient 1	Patient 2	Patient 3	Patient 1	Patient 2
Phenotype		NESCAV	NESCAV	NESCAV	NESCAV	NESCAV	PEHO	Congenital-onset cHSP	Congenital-onset cHSP	NESCAV	NESCAV
Age		5 yrs	3 yrs 5 mo	8 yrs	10 yrs	2 yrs 6 mo	15 yrs	6 yrs	21 yrs	2 yrs	6 yrs
Gender		F	F	M	F	F	F	M	F	F	M
Mutation, inheritance		c.296C>T, p.Thr99Met, de novo	c.296C>T, p.Thr99Met, de novo	c.296C>T, p.Thr99Met, de novo	c.296C>Tp.T99M de novo	c.296C>T p.T99M de novo	c.296C4T, p.(T99)M	c.296C>T, de novo	c.296C>T, de novo	c.296 C>T	c.296 C>T
Gestation, delivery period		UTI, HT	N/A	Normal	N/A	N/A	Minimal fetal movements, otherwise normal	N/A	N/A	N/A	N/A
Age of onset		Infancy (6 mo)	N/A	Infancy (8 mo)	Infancy	Infancy	Neonatal period	Infancy (6 mo)	Neonatal period	N/A	N/A
First abnormalities		Developmental delay	N/A	Developmental delay, infantile hypotonia	N/A	N/A	Infantile hypotonia, visual inattentiveness	N/A	N/A	N/A	N/A
Global development delay		Profound	Profound	Profound	Moderate	Profound	+	+	+	Profound	Profound
Ambulation		Non-ambulatory	N/A	N/A	Walks independently	Non-ambulatory	N/A	N/A	N/A	Non-ambulatory	Non-ambulatory
Speech		Simple syllables	N/A	N/A	Few words	Non-verbal	Non-verbal	Anarthria	Anarthria	N/A	N/A
Scoliosis		-	N/A	N/A	N/A	N/A	+	-	+	-	-
Hip subluxation		-	N/A	+	N/A	N/A	N/A	N/A	N/A	N/A	N/A
Microcephaly		-	N/A	-	-	+	+	+	-	+	+
Dysmorphic facial features		-	N/A	-	N/A	N/A	+	N/A	N/A	N/A	N/A
Growth deficit		-	N/A	GH deficit	N/A	N/A	+	N/A	N/A	N/A	N/A
Other abnormalities		-	N/A	↑ Aminotransferases, OSA, neurogenic bladder	-	-	Partial precocious puberty	-	-	-	-
Epilepsy, seizures		Polymorphic seizures	-	Generalized tonic–clonic convulsion at 4 yrs	-	Myoclonic seizures	Myoclonic seizures with infantile spasms	Epileptic spasms and focal seizures	Epileptic spasms and focal seizures	-	Tonic, myoclonic, generalized tonic–clonic seizures
EEG		Normal	-	Diffuse spikes	N/A	N/A	Hypsarrhythmia	Bilateral central abnormalities	Multifocal abnormalities		N/A
Antiepileptic treatment		Valproic acid, vigabatrin, levetiracetam, lacosamide (treatment was stopped at 2.5 yrs)	-	N/A	N/A	N/A	Vigabatrin, pyridoxine followed by lamotrigine	N/A	N/A	N/A	N/A
Spastic paraplegia		+	+	+	Spastic paraparesis	Spastic paraparesis	+	Spastic tetraparesis	Spastic tetraparesis	Spastic paraparesis	- *
Axial hypotonia		+	+	+	N/A	+	+	-	-	+	+
Cerebellar signs			N/A	Nystagmus	-	Mild ataxia	N/A	-	-	-	-
Neuropathy		+	N/A	N/A	-	-	N/A	Axonal sensory-motor neuropathy	Axonal sensory-motor neuropathy	N/A	N/A
Optic neuropathy		-	N/A	+	-	+	+	-	+	-	+
Cortical visual impairment		-	N/A	N/A	N/A	N/A	N/A	- **	+	+	+
Other neurological dysfunction		-	N/A	-	-	-	-	Extrapyramidal signs	-	-	Adventitious movements
MRI		Normal									
	Cerebellar atrophy	-	+ (Vermis)	+ (Vermis)	+	+	+	+	+	N/A	N/A
	Cerebral atrophy	-	-	+	+	+	-	-	+	N/A	N/A
	Dilatation of lateral ventricles	-	-	+ (Mild)	-	-	+	-	-	N/A	N/A
	Thin corpus callosum	-	-	+	-	-	+	+	+	N/A	N/A
Course		Progressive	-	Progressive	N/A	N/A	-	Progressive	Progressive	Progressive	Progressive

UTI—urinary tract infection, HT—hypothyroidism, GH—growth hormone, OSA—obstructive sleep apnea, * hyperreflexia was present, ** hypovision.

**Table 6:** Patient 2 with mutation c.38G>A and available literature data [7].

Patient	Our Patient 2	Tomaselli (2017) [7]
Mutation	2:240797715-C>T; c.38G>A; p.(Arg13His)	(c.38G>A, p.R13H)
Gender	Female	Male
Age	10 yrs	20 yrs
Family history	Normal	Normal
Birth	Normal	Normal
Onset of abnormalities	2 yrs	Early childhood
First abnormalities	Gait abnormalities, delayed speech	Delayed motor milestones
Psychomotor function	Walking at 24 months	ASD, ADHD
Epilepsy	No	No
Spasticity	Yes	At a later age, more severe in LL
Neuropathy	No	Reduced vibration sensation to the ankles
Nerve conduction velocity study	Slowing velocity in the lower limbs	Length-dependent sensory and motor axonal neuropathy with signs of chronic denervation in the lower limbs

## Data Availability

The data presented in this study are available on request from the corresponding author. The data are not publicly available due to ethical reasons.
